# Fellow-Workers and the Roll of Honour

**Published:** 1915-06-19

**Authors:** 


### ROUND THE WORLD.

[The Editor invites Early Information and Contributions Marked "Fellow-Workers."]

### Fellow-Workers and the Roll of Honour.

THE Queen Mary's Convalescent Auxiliary Hospitals, of which the consulting staff was referred to in these Notes last week, have been organised at Roehampton House and Dover House, near Barnes. These institutions will be especially devoted to the needs of sailors and soldiers in the convalescent stages who have lost limbs during the war, and some 300 of these unlucky men will be accommodated.

MR. ROBERT JONES, F.R.C.S. Edin. and Irel., lately appointed consulting surgeon to the Queen Mary's Convalescent Auxiliary Hospitals, is a well-known North Country surgeon and authority on orthopædics, whose work has been referred to in these notes on former occasions. A prominent member of the staff of Liverpool Royal Infirmary and teacher of surgery in the University there, this distinguished specialist has done much to further the development of modern methods of treating deformities and diseases of the bones and joints.

MR. THOMAS HORROCKS OPENSHAW, M.B., B.S., Durh., F.R.C.S. Eng., also on the staff of the Barnes war institutions, is one of the senior surgeons at the London Hospital, where he has for many years taken great interest in the orthopædic department, and is also on the staff of the Royal National Orthopædic and Poplar Accident Hospitals. He has made important contributions to the literature of orthopædic surgery.

THE Surgeon-Commandant of the Allied Forces Base Hospital, formerly at Boulogne and in future to be carried on at Etaples, is Lieutenant-Colonel William Ernest Miles, who in civil life has occupied a leading position amongst London surgeons for many years. Qualifying from St. Bartholomew's in 1891, and subsequently taking the F.R.C.S. Eng., Lieutenant-Colonel Miles held resident posts at the Radcliffe Infirmary, Oxford, the Metropolitan Hospital, and St. Mark's Hospital; then, settling in private surgical practice, he became attached to the Cancer Hospital and to the Gordon Hospital for Fistula, being now surgeon to both these institutions.

ANOTHER Canadian medical man who has lost his life in recent fighting was Major W. P. Dillon, of the No. 2 Canadian General Hospital, who died of wounds.

SURGEON EDWARD G. SCHLESINGER, R.N., who has been wounded whilst serving in the Dardanelles campaign, was entering upon a career of great promise at Guy's Hospital when the war broke out, and he left to take up military duties. Entering the medical school with a science scholarship in 1906, he won the Michael Harris prize in anatomy and took the B.Sc. Lond. with first-class honours two years later. Mr. Schlesinger qualified in 1911, taking the "Conjoint" diploma and the M.B., B.S. Lond. degree, being awarded honours at the examination for the latter; whilst subsequently he held the post of house surgeon at his hospital, and proceeded to the F.R.C.S. Eng. Last summer his keen work was rewarded by his appointment to a surgical registrarship and demonstratorship in anatomy at Guy's, but owing to the turn of events was unable to take up the duties of those posts.

NOTABLE researches have lately been in progress at St. Thomas's Hospital concerning the nature of the germs commonly found in infected wounds. Working with clinical material from that institution, as well as from the 2nd London General Hospital, Dr. Leonard S. Dudgeon—in collaboration with Dr. A. D. Gardner and Mr. F. Bawtree—have arrived at certain important conclusions, on which they have just made a preliminary report to the *Lancet* (June 12, 1915). The expenses of this research were partly defrayed by a grant from the National Research Committee.

DR. LEONARD STANLEY DUDGEON, F.R.C.P. Lond., is the leading spirit in bacteriological research at St. Thomas's, where he occupies the posts of bacteriologist and director of the pathological laboratory, as well as of the well-equipped Louis Jenner Clinical Laboratory. Qualifying in 1899 as a St. Thomas's Hospital student, he quickly evinced special aptitude for scientific work, and as a Louis Jenner student in pathology became a prominent figure among the junior investigators then at that institution. Subsequently he was for a time lecturer in general pathology at the London School of Medicine for Women. Dr. Dudgeon, whose work has illumined more than one department of morbid histology and anatomy, has delivered the Erasmus Wilson lectures at the Royal College of Surgeons, and also the Croonian and Horace Dobell lectures at the Royal College of Physicians.

IN an interesting letter to the *St. Bartholomew's Hospital Journal*, Fleet-Surgeon Walter Kempson Hopkins, R.N., gives a summary of his experiences on H.M.S. *Fearless*, which have, it seems, included various dealings with destroyers and submarines. Mr. Hopkins qualified L.R.C.P., M.R.C.S. as a "Bart.'s" student in 1896.

MAJOR ELWIN HARRAL THOMAS NASH, who has been granted his present rank temporarily whilst serving with the Lord Derby War Hospital, has occupied important public health appointments, including that of medical officer of health, school medical officer, and bacteriologist for Wimbledon. A student of Manchester and St. Thomas's Hospital, he qualified L.R.C.P., M.R.C.S. in 1896 and took the D.P.H. Vict. eight years later. Last year he published a work on "School Lighting."

MAJOR RICHARD GUNDRY ROWS, who has lately been on the staff of the Lancaster County Asylum, has been granted his present temporary commission and rank on appointment to the Moss-side Military Hospital. A University College Hospital man, who qualified in 1891, and subsequently became an M.D. Lond., Major Rows took up psychiatry as a specialty early in life, and held appointments at the Birmingham City Asylum and at the County Asylum at Prestwich. He has devoted much time to pathological research, and published communications on infective processes affecting the central nervous system.

LIEUTENANT J. C. JEFFERSON, R.A.M.C. (T.F.), surgical registrar to the Manchester Royal Infirmary, and Lieutenant O. H. Blacklay, R.A.M.C. (T.F.), who have been working at the Cairo Military Hospital, have communicated an account of some of their experiences to the *British Medical Journal* (June 12, 1915).

